# Functional study of a novel missense single‐nucleotide variant of NUP107 in two daughters of Mexican origin with premature ovarian insufficiency

**DOI:** 10.1002/mgg3.345

**Published:** 2018-01-24

**Authors:** Yu Ren, Feiyang Diao, Sunita Katari, Svetlana Yatsenko, Huaiyang Jiang, Michelle A. Wood‐Trageser, Aleksandar Rajkovic

**Affiliations:** ^1^ Department of Obstetrics, Gynecology, and Reproductive Sciences Magee‐Womens Research Institute University of Pittsburgh Pittsburgh PA USA; ^2^ State Key Laboratory of Reproductive Medicine Center of Clinical Reproductive Medicine Nanjing Medical University Nanjing China; ^3^ Division of Reproductive Endocrinology and Infertility Magee‐Womens Hospital of UPMC Pittsburgh PA USA; ^4^ Department of Pathology University of Pittsburgh Pittsburgh PA USA; ^5^ Department of Human Genetics University of Pittsburgh Pittsburgh PA USA

**Keywords:** CRISPR, hypergonadotropic hypogonadism, infertility, Nup107, ovary

## Abstract

**Background:**

Hypergonadotropic hypogonadism (HH) is a genetically heterogeneous disorder that usually presents with amenorrhea, atrophic ovaries, and low estrogen. Most cases of HH are idiopathic and nonsyndromic. Nucleoporin 107 (*NUP107*), a protein involved in transport between cytoplasm and nucleus with putative roles in meiosis/mitosis progression, was recently implicated as a cause of HH. We identified a *NUP107* genetic variant in a nonconsanguineous family with two sisters affected with primary amenorrhea and HH, and generated a mouse model that carried the human variant.

**Methods:**

We performed a high‐resolution X‐chromosome microarray and whole exome sequencing on parents and two sisters with HH to identify pathogenic variants. We generated a mouse model of candidate *NUP107* variant using CRISPR/Cas9.

**Results:**

Whole exome sequencing identified a novel and rare missense variant in the *NUP107* gene (c.1063C>T, p.R355C) in both sisters with HH. In order to determine functional significance of this variant, we used CRISPR/Cas9 to introduce the human variant into the mouse genome. Mice with the homolog of the R355C variant, as well as the nine base pairs deletion in *Nup107* had female subfertility.

**Conclusions:**

Our findings indicate that *NUP107* R355C variant falls in the category of variant of unknown significance as the cause of HH and infertility.

## 1 INTRODUCTION

Hypergonadotropic hypogonadism (HH), also known as premature ovarian insufficiency, affects 1–4% of women and is defined as a cessation of menses prior to age 40, with elevated follicle‐stimulating hormone (FSH) and low serum estradiol levels. Women with HH can present with amenorrhea (primary or secondary), hypogonadism, and hypoestrogenic symptoms (i.e., hot flashes, vaginal dryness, premature osteoporosis). HH is genetically heterogeneous, with few genes identified, and can be idiopathic and nonsyndromic or part of a genetic syndrome. Pathogenesis of HH includes abnormalities of the X chromosome or autosomes and can also be due to autoimmune, infectious, and environmental causes (LM, 2009). For a subset of idiopathic cases, a genetic basis has been shown, and FMR1 (MIM 309550), FSHR (MIM 136435), PMM2 (MIM 601785), GALT (MIM 606999), AIRE (MIM 607358), and FOXL2 (MIM 605597) are the genes currently recommended for clinical genetic testing (Practice Committee of American Society for Reproductive Medicine, 2008).

Recently, a nucleotide missense variant in *NUP107* (c.1339G>A, p.D447N, MIM 607617) was associated with HH and XX female gonadal dysgenesis in a consanguineous Palestinian family (Weinberg‐Shukron et al., 2015). Nucleoporins assemble to form nuclear pore complexes (NPCs), which are embedded throughout the double‐membrane nuclear envelope. NPCs are regarded as the gatekeepers of the nucleus and tightly control all nucleocytoplasmic transport (Kabachinski & Schwartz, 2015). Nucleoporin 107 kDa (*NUP107*) is evolutionarily conserved, and it is a key scaffold protein in NPC assembly, as a part of the *Nup107–Nup160* subcomplex (Walther et al., 2003; Belgareh et al., 2001).

Here we report a new *NUP107* (c.1063C>T, p.R355C) variant, discovered in two sisters who were diagnosed with primary amenorrhea and HH. The R355 amino acid is conserved across species, and this variant is present at low frequency in heterozygous state (1.81 × 10^−5^) in the gnomAD database (Lek et al., 2016). The R355 amino acid is predicted to form a salt bridge with D447, which is important to stabilize overall NUP107 protein structure (Weinberg‐Shukron et al., 2015). Classification of nucleotide variants for clinical use requires either epidemiologic or in vitro and in vivo assays that corroborate or disprove pathogenicity of the variant. We generated a mouse model to determine functional significance of the *NUP107* (c.1063C>T, p.R355C) variant.

## 2 METHODS

### 2.1 Study approval

The recruitment of fertile women from Magee‐Womens Hospital was approved by the IRB of the University of Pittsburgh. Written informed consent was obtained from all participating subjects. All experimental and surgical procedures complied with the Guide for the Care and Use of Laboratory Animals were approved by the Institutional Animal Care and Use Committee at the University of Pittsburgh.

### 2.2 Whole exome sequencing

We used HaloPlex exome capture kit (Agilent) to capture exons and splice sites, and whole exome sequencing (WES) was performed on an Illumina HiSeq 2500. Sequence reads were mapped to the human reference genome (GRCh37/h19) using Burrows–Wheeler Aligner version 0.7.3a MEM (maximal exact match). Local realignment around insertions and deletions and recalibration of read base quality were conducted with Genome Analysis Toolkit (GATK) version 2.8. GATK Haplotype Caller was used for calling variants.

### 2.3 Copy number variation

Microarray hybridization experiments and analyses were performed according to the manufacturer's protocol using pooled female DNA (G152, Promega) as the reference and as described previously (Yatsenko et al., 2015). Copy number variants were detected using the aberration detection method 2 (ADM‐2) algorithm and displayed by Cytogenomics v2.5.8 software (Agilent) and compared to a database of apparently copy number variants obtained from normal control individuals (DGV, http://dgv.tcag.ca) and other public databases of copy number variants (ClinGen CNVs, http://www.clinicalgenome.org; DECIPHER, https://decipher.sanger.ac.uk).

### 2.4 Generation of transgenic mouse model

We used CRISPR/Cas9n to introduce a corresponding human missense variant into the mouse *Nup107* (c.1066C>T, p.R356C; Figure 2). A pair of single guide RNAs (sgRNAs)—sgRNA1 and sgRNA2—targeting exon 12 of the *Nup107* gene were designed by an Optimized CRISPR Design tool (http://crispr.mit.edu/) (Figure 2a). The desired variant site falls inside of the sgRNA‐targeted region. sgRNAs were cloned into PX461 vector, which contains the D10A Cas9 mutant (Cas9n) sequence and GFP tag. One 191‐nt single‐stranded oligodeoxynucleotide (ssODN) carrying the single nucleotide change was synthesized to be the template of homology‐directed repair (Figure 2b). It contains flanking sequences of 60 nucleotides on each side that are homologous to the regions of intron 11/exon 12 and exon 12/intron 12, respectively (Figure 2b). The function of the sgRNA pair and ssODN was validated by delivering a mix of Cas9n:sgRNA1 + Cas9n:sgRNA2 + ssODN into neuro‐2a cells (data not shown). The Cas9:sgRNA vectors and ssODN were injected into the zygote to generate the transgenic mice. After overnight culture, surviving two‐cell embryos were transferred to ICR pseudopregnant recipients. The successful introduction of the missense variant c.1066C>T was verified by Sanger sequencing (Figure 2c). Other regions of the exon 12 were not changed compared to the wild‐type sequences, except a synonymous variant at c.1068T>C, which was intentionally modified for creating a HhaI recognition site, GCGC, for the purpose of mouse genotyping (Figure 2c,d).

### 2.5 Statistics

Data were analyzed using Excel and are presented as *M* ± *SEM*. Results were analyzed using two‐tailed Student's *t* test.

## 3 RESULTS AND DISCUSSION

**Figure 1:**
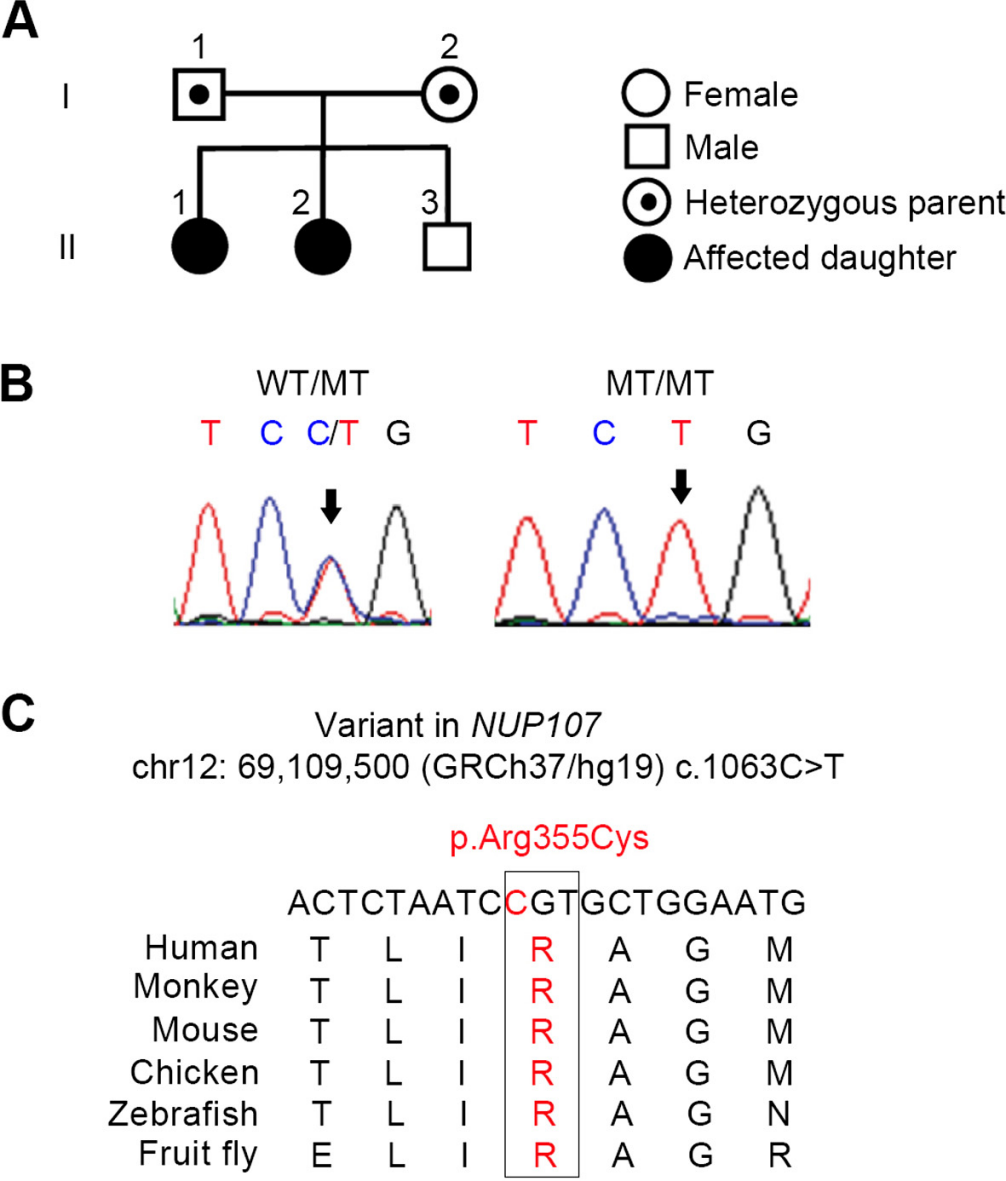
*NUP107* variant (c.1063C>T, R355C) is present in two daughters from a nonconsanguineous family. (a) Family members are designated by Arabic numerals. Horizontal lines between individuals represent marriage. Vertical lines represent lineage. I‐1 and I‐2, II‐1 and II‐2 underwent whole exome sequencing. (b) Representative chromatograms resulting from Sanger sequencing following PCR surrounding the *NUP107* variant (chr12:69,109,500 GRCh37/hg19). Parents who are heterozygous for the *NUP107* c.1063C>T variant show overlapping C and T peaks (WT/MT). Daughters homozygous for the variant have a single T peak (MT/MT). (c) The NUP107 residue R355 is highly conserved among species ranging from fruit flies to humans. The single‐nucleotide variant of C (red) to T is predicted to change the NUP107 355 amino acid residue from arginine (R) to cysteine (C)

A healthy couple of Mexican descent had two daughters diagnosed with primary ovarian insufficiency (see pedigree in Figure 1a). There is no known history of consanguinity in the family. Both sisters presented with primary amenorrhea and delayed puberty at 17 and 15 years of age. They have an older brother who is healthy with normal pubertal development. Both sisters had a normal 46, XX karyotype, screened negative for Fragile‐X carrier status, had normal thyroid function and glucose tolerance, and had negative thyroid and adrenal antibody screens (Table S1). As expected for HH, their FSH and LH levels were high, with low levels of estradiol and pelvic ultrasound revealed atrophic ovaries (Table S1). There was no significant exposure (radiation, chemotherapy) or surgical history to suggest iatrogenic cause for the hypogonadism. Their mother attained menarche at age 12, and reports having normal pubertal development and regular menstrual cycles. She had three uneventful full‐term pregnancies. Her medical history was significant for hypothyroidism, diagnosed at age 28. There was no known family history of delayed puberty, infertility, or premature menopause.

We initially utilized a custom‐designed oligonucleotide CGH microarray (Yatsenko et al., 2015) to examine for pathologic genomic imbalances involving both X‐linked and autosomal genes associated with HH. This oligonucleotide‐based 180K CGH microarray has been designed to achieve high‐resolution detection of losses and gains for 397 gonadal genes which include known and candidate genes implicated in gonadal dysgenesis, HH, steroidogenesis, and ovarian and testicular differentiation derived from studies in humans and animal models (Dangle et al., 2017). In addition to covering the autosomal regions of the genome, this microarray platform densely covers X chromosome to achieve a resolution of ~0.3–3 kb. No pathologic copy number variants were identified in the two affected sisters.

WES was performed on the parents and affected daughters to identify nucleotide variants that may account for the idiopathic and nonsyndromic HH. Variants were filtered for quality and significance as previously reported (AlAsiri et al., 2015). We filtered for nonsynonymous variants (in exons or splice sites) that are presumed to be damaging, with a minor allele frequency of <5% and assumed recessive inheritance given the pedigree. Variants in four genes (*ABCD2*,* ITPR2*,* NUP107*,* THAP2*) satisfied the above criteria (Table S2). We further annotated these variants based on the pathogenicity (Richards et al., 2015) gene expression profile, protein function, its conservation status, and any available data from animal experiments. ABCD2 was expressed in a number of tissues, including ovary, while *NUP107* RNA is highly expressed in the ovaries and oocytes as per BioGPS RNA profiling in mouse tissues (Wu et al., 2009). In the human embryonic ovaries, *NUP107* was highly expressed while *ABCD2* was not detectable (data not shown). *ABCD2* was ruled out as a candidate because mouse knockouts had cerebellar and sensory ataxia without infertility (Ferrer et al., 2005). We focused our attention on the *NUP107* (c.1063C>T, p.R355C) variant as a candidate pathogenic variant, present in homozygous state in the affected daughters. The *NUP107* c.1063C>T variant was verified in the family by Sanger sequencing (Figure 1b). The c.1063C>T variant was predicted to cause amino acid change p.R355C, at a highly conserved residue in exon 12 of *NUP107* (Figure 1c), and corresponds to a mouse R356 residue.

**Figure 2:**
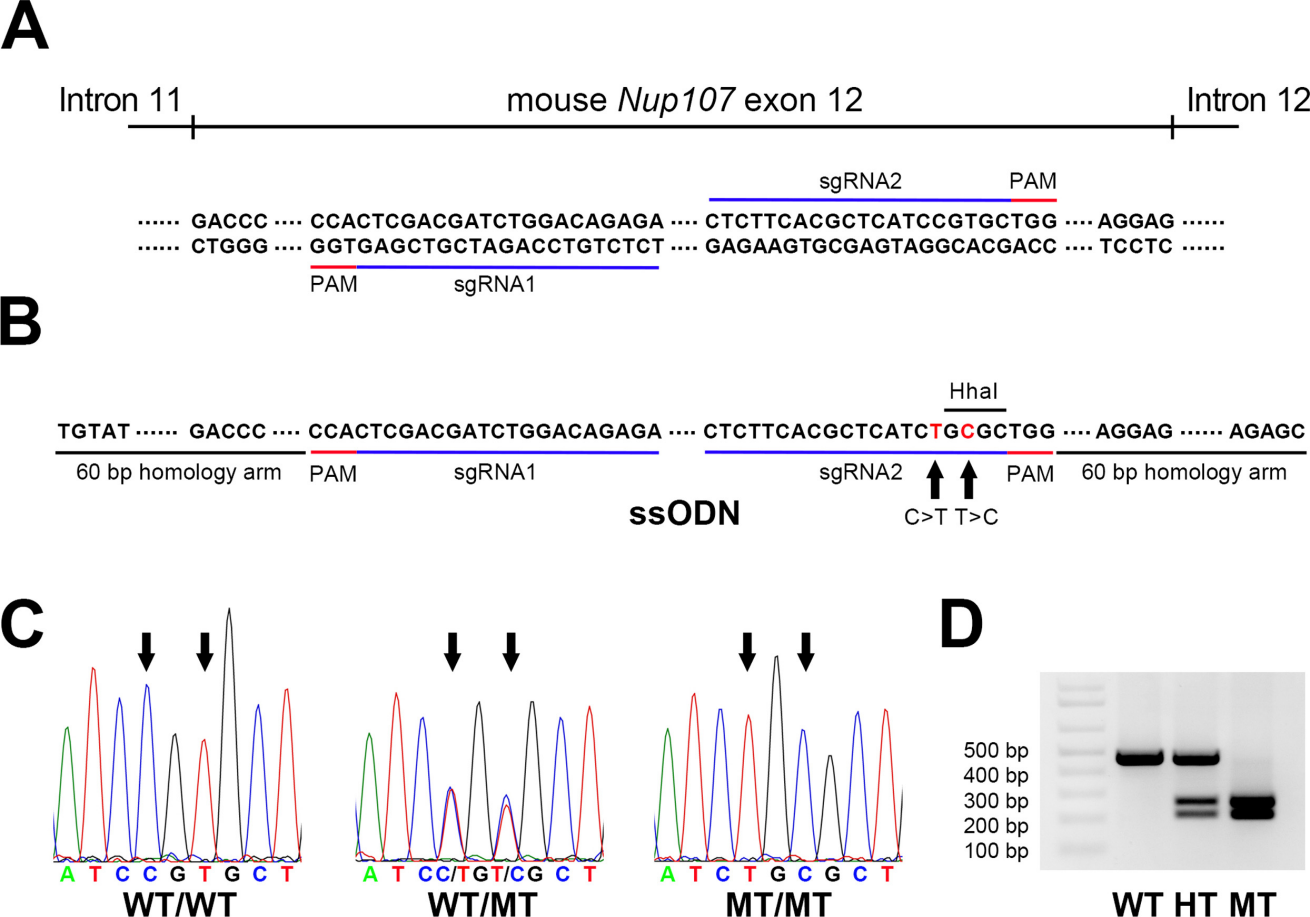
CRISPR/Cas9 mediated modeling of the human *NUP107* c.1063C>T variant in mice by introducing a missense variant (c.1066C>T) into mouse *Nup107* gene. (a) Sequences of a pair of sgRNAs (underlined in blue lines) that target *Nup107* exon 12. The PAM sequences are underlined in red. (b) The sequence of ssODN bearing the single‐nucleotide variant of c.1066C>T, which changes the coded amino acid from arginine to cysteine. A synonymous variant at c.1068T>C was also introduced to create a HhaI recognition site, GCGC, to aid in mouse genotyping. (c) Chromatograms from Sanger sequencing are shown for homozygous wild‐type (WT/WT), heterozygous (WT/MT), and homozygous variant (MT/MT). Arrows indicate the location of missense variants. (d) Genotyping the wild‐type (WT), heterozygous (HT), and homozygous variant (MT) mice. The variant PCR product will be cut into two bands (260 and 210 bp) by HhaI if there is a variant allele

**Figure 3:**
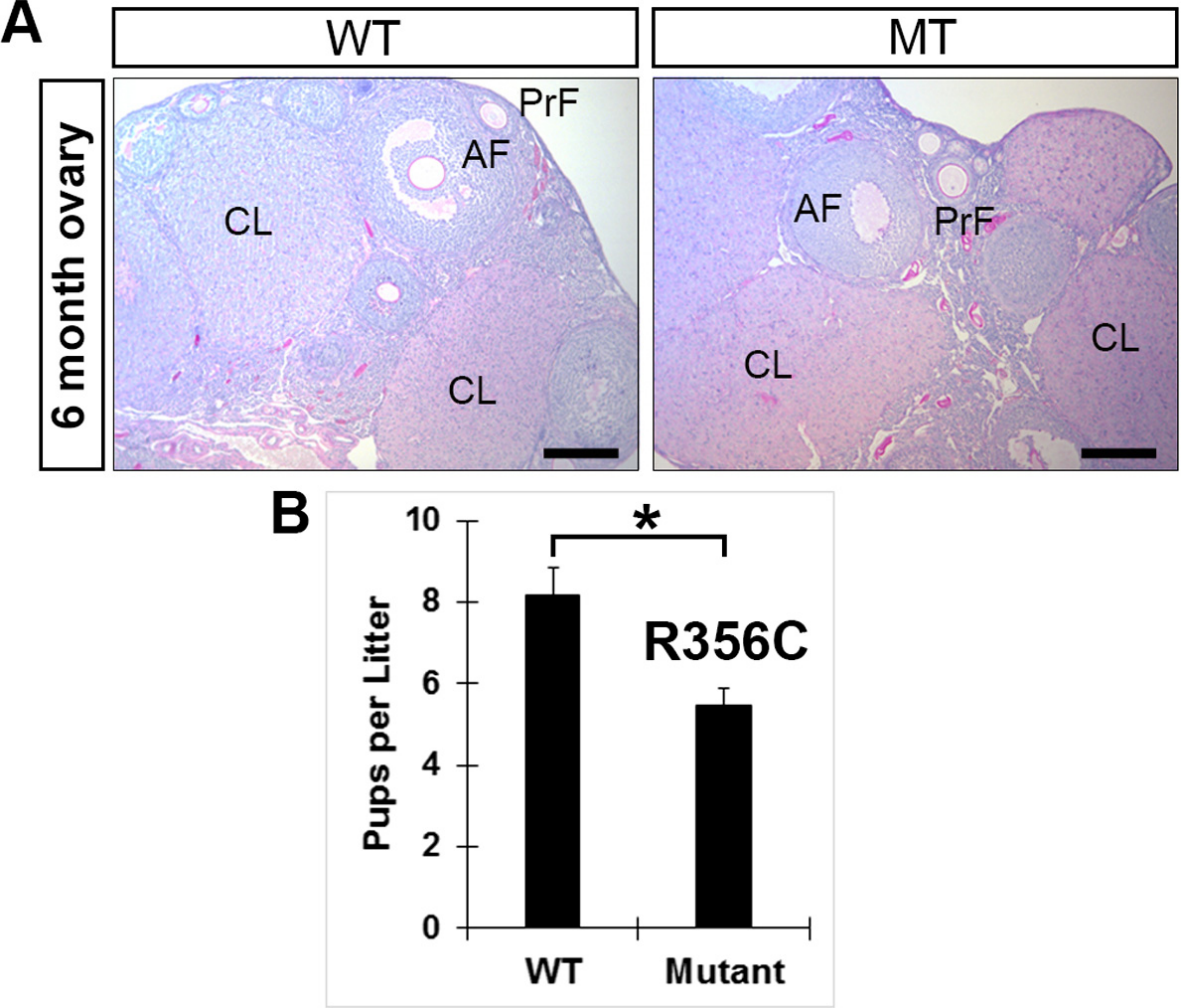
The *Nup107* c.1066C>T, p.R356C variant causes subfertility without affecting ovarian development. (a) Periodic acid–Schiff (PAS) staining in 6‐month‐old wild‐type (WT) and *Nup107* ovaries homozygous for the variant (MT). PrF, primary follicle; AF, antral follicle; CL, corpus luteum. Scale bars: 200 μm. (b) Fertility tests of *Nup107* females homozygous for the R356C variant (MT), and mated with wild‐type mice over a period of 12 months (*n* = 10 per group) show significant difference in pups per litter when compared to the wild‐type (WT) mating. Error bars indicate the *M* ± *SD*. Two‐tailed Student's *t* test was used to calculate *p* values (*p* < .001)

We introduced the human missense *NUP107* variant into the mouse using CRISPR/Cas9n to generate a mouse that carries *Nup107* (c.1066C>T, p.R356C) variant (Figure 2). A total of 18 founder animals were obtained. One founder carried homozygous missense variant c.1066C>T verified by Sanger sequencing (Figure 2c). Other regions of the exon 12 were not changed compared to the wild‐type sequences, except a synonymous variant at c.1068T>C, which was intentionally modified for creating a HhaI recognition site, GCGC, for the purpose of mouse genotyping (Figure 2c,d). Female founders were mated with wild‐type males and F2 generation used for fertility studies in order to eliminate mosaicism. We mated F2 heterozygotes and confirmed homozygous *Nup107* ^*R356C*^ genotypes by Sanger sequencing. Homozygous *Nup107* ^*R356C*^ founder females were subfertile when mated to wild‐type males over a period of 12 months (Figure 3b). The mean number of pups per litter was 8.2 ± 3.1 for wild‐type control, and 5.5 ± 2.3 for *Nup107* ^*R356C*^ homozygotes mated with wild‐type males, a 33% reduction (*p* < .001). *Nup107* p.R356C heterozygous females fertility did not significantly differ from wild‐type mice. There was no significant difference between wild‐type and *Nup107* ^*R356C*^ females in the number of litters per month. Males homozygous for the *Nup107* ^*R356C*^ variant were fertile. Homozygous females had grossly normal ovaries and histology showed a normal range of follicles when compared with the wild‐type mice (Figure 3a). Males homozygous for the *Nup107* ^*R356C*^ were also fertile and testes histology was not significantly different from wild‐type mice.

A recent study identified a consanguineous family of Palestinian origin, in which four females exhibited HH (Weinberg‐Shukron et al., 2015). These investigators used homozygosity mapping and WES to identify a recessive missense variant in nucleoporin 107 (*NUP107*, c.1339G>A, p.D447N). This variant segregated with the phenotype and was not present in available databases or in 150 healthy ethnically matched controls. The investigators then used transgenic rescue of *Drosophila* females bearing the human homolog, *Nup107* D364N variant, to show subfertility phenotype, with a marked reduction in progeny, morphologically aberrant eggshells, and disintegrating egg chambers, indicating defective oogenesis. Their results indicated a role for *NUP107* in ovarian development and suggest that nucleoporin defects may play a role in HH. It is interesting to note that *NUP107* positively charged R355 and K278 form salt bridges with negatively charged D447 (Weinberg‐Shukron et al., 2015). These salt bridges are crucial in maintaining the stability of the whole NUP107 protein and variant of a corresponding site of human *NUP107* D447 variant in *Drosophila* reduces female fertility, presumably due to disrupted salt bridges (Weinberg‐Shukron et al., 2015). In the variant of arginine to cysteine, positive charge is lost; however, mouse NUP107 protein function seems not to be affected.

Our WES generated several plausible variants of unknown significance. *Nup107* (c.1066C>T, p.R355C) variant was investigated further by introducing it in the mouse. Mice are good models for human reproductive tract disease, and replicate the human phenotype of HH for many genes studies such as *Fshr* and *Sohlh1* (Dierich et al., 1998; Aittomaki et al., 1995; Katari et al., 2015; Bayram et al., 2015). Missense variants, which result in single or few nucleotide changes that convert one amino acid to another are much more common, but more difficult to functionally assess. Recent studies using CRISPR/Cas9 suggest that introduction of human variants into mice reveals functionally significant missense variants (Singh & Schimenti, 2015). Unlike our affected daughters that were infertile and had small ovaries, *Nup107* ^*R356C*^ female mice were subfertile and ovaries were not hypoplastic. The difference between subfertility and total infertility could be due to the basic difference between human and mouse reproductive systems. Mice have large litter sizes as compared to humans, and subfertility in mice may translate into total infertility in humans. Alternatively, *Nup107*, despite its conservation, may have different functions in the mouse ovaries as compared to the human.

We conclude that *Nup107* (c.1066C>T, p.R355C) variant falls in the category of variant of unknown clinical significance, and further human studies are needed for classification into benign or pathogenic category. This conclusion is based on the application of recent guidelines issued by the American College of Medical Genetics and Genomics and the Association for Molecular Pathology (Richards et al., 2015). Over 500 genes and their variants have been reported to disrupt hypothalamic–pituitary–gonadal axis and cause hypogonadism. Precise genetic diagnosis of such reproductive physiologic disturbances can lead to appropriate hormonal replacement, correction of underlying physiologic disturbance, and treatment of infertility. ClinGen and ClinVar entries are underrepresented in the description of pathogenic variants in infertility genes, despite over 300 entries in OMIM. Commercial and academic laboratories offer multiple gene panels for infertility, ovarian insufficiency, hypogonadism, and disorders of sexual differentiation. Misinterpretation of pathogenicity of such variants could lead to unnecessary medical intervention, either hormonal or surgical. Curation and appropriate interpretation of genetic variants is of great importance for the future of precision reproductive medicine.

## CONFLICT OF INTEREST

The authors have declared that no conflict of interest exists.
