# Supplementary material for: Functional study of a novel missense single‐nucleotide variant of NUP107 in two daughters of Mexican origin with premature ovarian insufficiency
Source: Mol Genet Genomic Med. 2018 Jan 24;6(2):276–81. doi: 10.1002/mgg3.345 (PMC5902394; doi:10.1002/mgg3.345)
Supplement: Supplementary file 1 [file MGG3-6-276-s001.docx]

Supplementary Tables

| **Table S1. Clinical and Laboratory Data** | | | |
| --- | --- | --- | --- |
| Clinical Data | Normal  range | II-1 | II-2 |
| Age at diagnosis |  | 17 | 15 |
| Presentation |  | Delayed puberty,  Primary amenorrhea | Delayed puberty  Primary amenorrhe |
| Height in inches |  | 157 | 152 |
| Weight in kgs |  | 53.8 | 43.5 |
| BMI | 18.9 -24.9 | 21.8 | 17 |
| Tanner stage-Breast | II-IV | 2 | 2 |
| FSH mIU/ml | 1-9.2^b^ | 96 | 122 |
| LH mIU/ml | 0.3- 29.4^b^ | 42 | 26.3 |
| Estradiol pg/ml | 30-300^c^ | <10 | <10 |
| TSH mIU/ml | 0.3-5^c^ | 2.96 | 3.37 |
| Free T4 ng/dl | 0.75-1.54^c^ | 1.3 | 1.6 |
| Adrenal antibody screen | Negative | Negative | Negative |
| Karyotype | 46 XX | 46XX | 46XX |
| Fragile- X screen | <44 CGG repeats | <44 | <44 |
| Pelvic Ultrasound | Uterus: 20-100cc  Ovaries: 6.6±0.19cc | Uterus- 5 cc , Right Ovary- 0.5cc, Left ovary- 0.5cc | U- 1.6 cc, Right Ovary- 0.75 cc, Left ovary- 0.3cc |
| All hormone measures provided were prior to hormone replacement therapy.  ^b^Mayo Clinic, Mayo Medical Laboratories, Mayo Clinic, Minnesota.  ^c^Reference range for adolescent girls. | | | |

**Table S2. Whole Exome Sequencing Variant Calls**

| Gene | Chr Position | Chr | Cyto band | Reference Nucleotide | Mutation Call | Inheritance pattern | Protein conservation (mouse: human) | Amino Acid Change |
| --- | --- | --- | --- | --- | --- | --- | --- | --- |
| *ABCD2* | 39979971 | 12 | q12 | A | A>T | Autosomal recessive SNV | 96% | 592I>K |
| *NUP107* | 69109500 | 12 | q15 | C | C>T | Autosomal recessive SNV | 96% | 355R>C |
| *ITPR2* | 26816744 | 12 | p11 | T | T>A | Autosomal recessive SNV | 97% | 529K>N |
| *THAP2* | 72070803 | 12 | q.21.1 | A | A>G | Autosomal recessive SNV | 94% | 201E>G |
